# Over-Eating

**Published:** 1888-12

**Authors:** 


### OVER-EATING.

It is a decree that while civilized man cannot live without dining, he might live a great deal longer without *so much* dining, or rather without dining so extensively. Sir Henry Thompson says that he has been compelled by facts to think that more mischief in the form of disease has accrued to civilized man from erroneous habits in eating than from the use of alcoholic drink, He also declared himself in doubt whether improper and inordinate eating were not as great a moral evil as inordinate drinking. Many of our best physicians say that the habit of over-eating is at the bottom of most troublesome diseases. Doubtless this habit is most often laid in childhood. How many mothers feed their babies as often as they cry, taking it for granted in the most imbecile manner that the baby cries for food, when more often the helpless little victim cries because it already has had too much food. When the stomach once becomes accustomed to being crowded with food, if the supply is cut short there is at first a gnawing sensation that is frequently mistaken for hunger. Persevere a little longer in your abstinence, and you will find yourself benefited by it.

